# Autologous platelet concentrates for facial rejuvenation

**DOI:** 10.1590/1678-7757-2022-0020

**Published:** 2022-09-05

**Authors:** Marília Afonso Rabelo BUZALAF, Flávia Mauad LEVY

**Affiliations:** 1 Universidade de São Paulo Faculdade de Odontologia de Bauru Departamento de Ciências Biológicas Bauru SP Brasil Universidade de São Paulo, Faculdade de Odontologia de Bauru, Departamento de Ciências Biológicas, Bauru, SP, Brasil.

**Keywords:** Platelet-rich plasma, Platelet-rich fibrin, Facial rejuvenation, Autologous platelet concentrates

## Abstract

Autologous platelet concentrates (APCs) are promising therapeutic agents in facial rejuvenation since they are a great source of cytokines, growth factors and other biologically active substances. Obtained from the patient’s blood, they have the advantages of reducing immunological reactions, making the procedure safer, well tolerated, with minimal adverse effects and lower cost. Currently, they are used for facial rejuvenation both in combination with microneedling and in mesotherapy techniques, as well as to treat facial acne scars, melasma and wounds after laser ablative treatments. This review summarizes current knowledge on the use of APCs, ranging from basic concepts related to their composition and mechanisms of action to up-to-date information on their clinical efficacy.

**Methodology:**

MEDLINE (PubMed) was searched from inception through 2021 for English language publications on APCs for facial rejuvenation.

**Results:**

A total of 100 files were found. Based on the available literature, APCs for skin rejuvenation are safe and well tolerated. The most studied product is the first-generation material, platelet-rich plasma (PRP).

**Conclusions:**

The results are in general favorable, but the quality of the studies is low. The second and third generation products, platelet-rich fibrin (PRF) and injectable platelet-rich fibrin (i-PRF), respectively, are easier to be obtained and, at least *in vitro* , seem to induce greater collagen production than PRP, especially under lower relative centrifugation forces, but to date only a few clinical trials evaluating these products exist. More high-quality trials with appropriate follow-up are necessary to provide adequate evidence that may help to improve the treatment regimens with APCs. Many aspects should be considered when designing clinical trials to evaluate APCs, such as the patients’ characteristics that best predict a favorable response, the optimal number of sessions and the interval between them, the characteristics of the studies and the development of better instruments to evaluate skin aging.

## History and evolution of autologous platelet aggregates for facial rejuvenation

Autologous platelet concentrates are promising therapeutic agents in regenerative medicine since they are a great source of cytokines, growth factors and other biologically active substances. They are increasingly being used in distinct areas of Dentistry, such as periodontal surgery and orofacial harmonization and in Medicine, such as orthopedics, surgery, sports medicine and aesthetic dermatology. The use of autologous preparations has the advantages of reducing immunological reactions and disease transmission, making the procedure safer, well tolerated, with minimal adverse effects and lower cost, since the material is obtained from the patient after the collection of peripheral blood and its centrifugation.

The origin of the therapy comes from transfusiology, where platelet concentrates are used to treat thrombocytopenia.^1^ In 1954, for the first time, the term “platelet-rich-plasma” (PRP) was employed by Kingsley^2^ (1954), when referring to platelet concentrates for transfusion. The first clinical demonstration that autologous platelet concentrates promoted healing when used locally was reported by Knighton, et al.^3^ (1986). At that time, the preparation used was called “Platelet-derived wound healing factors” (PDWHF). The use of the term “platelet-rich plasma” (PRP) in the context of regenerative dentistry/medicine began with Marx, et al.^4^ (1998), when the product was used in maxillofacial surgery for bone reconstruction.

PRP has been used for facial rejuvenation, with modest improvement in facial appearance, skin texture and wrinkles.^5^ However, its preparation is difficult, as it requires double centrifugation.^6^ In addition, the anticoagulants required can impair healing by inhibiting the coagulation process.^7^ To overcome some of these limitations of PRP, platelet-rich fibrin (PRF), a platelet concentrate called the “second generation”, was developed by Choukroun, et al.^8^ (2001). PRF is obtained through a single centrifugation, without the need for anticoagulants, being, therefore, fully autologous. The resulting product contains different cell types (platelets, leukocytes, erythrocytes), an extracellular fibrin matrix and a range of bioactive molecules (predominantly growth factors). Depending on the collection tube and on the centrifugation protocol used, PRFs in liquid or solid gel forms can be obtained. Solid forms, obtained with the use of glass tubes, have been widely used in maxillofacial surgery^7^ and plastic surgery,^9^ with benefits for bone and soft tissue regeneration, infection control and patient satisfaction.

**Figure 1:**
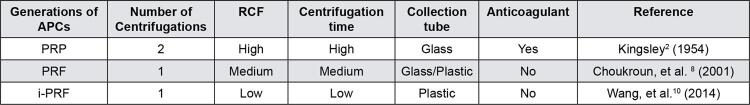
Differences between the distinct generations of autologous platelet concentrates *APCs – autologous platelet concentrates; PRP – platelet-rich plasma ; PRF – platelet-rich fibrin ; i-PRF – injectable platelet-rich fibrin ; RCF – relative centrifugation force.

In 2014, a fluid, injectable form of PRF (called i-PRF) was developed by modifying the relative centrifugation force (RCF).^10^ By decreasing centrifugation speed and time and using plastic tubes (to reduce clotting time), fibrin clotting could be slower in the initial time periods, generating a product containing fibrinogen and thrombin that remains fluid for about 20 minutes after centrifugation, before the formation of fibrin. This makes it an appropriate material to be used in facial rejuvenation. Figure 1 summarizes the main differences among the distinct generations of autologous platelet concentrates.

Currently, autologous platelet concentrates are used for facial rejuvenation both in combination with microneedling (drug delivery), and in mesotherapy techniques.^5 , 11 , 12^

In addition to being used for skin rejuvenation, platelet concentrates have also been used to treat facial acne scars,^13^ melasma,^14^ as well as wounds after laser ablative treatments,^5 , 11^ as they lead to more efficient and fast healing. The market for PRP presented an impressive growth from around $ 45 million in 2009 to $120 million in 2016. It is expected to exceed $4.5 billion by 2024.^15^

This review summarizes current knowledge on the use of autologous platelet concentrates for facial rejuvenation, ranging from basic concepts related to their composition and mechanisms of action to up-to-date information on their clinical efficacy.

## Methodology

MEDLINE (PubMed) was searched on August 25, 2021 for English language publications on autologous platelet concentrates for facial rejuvenation. Search terms were: [ *facial rejuvenation* AND ( *platelet rich plasma* OR *platelet rich fibrin* OR *injectable platelet rich fibrin* OR *iPRF* OR *PRF* OR *PRP* )]. A total of 100 files were found. Titles, abstracts and full-texts were independently screened by two reviewers (MB and FM). One file was excluded because it was an editorial. Other 28 files were articles unrelated to facial rejuvenation and were also excluded.

## Results and discussion

### Composition and mechanisms of action of autologous platelet concentrates

**Figure 2:**
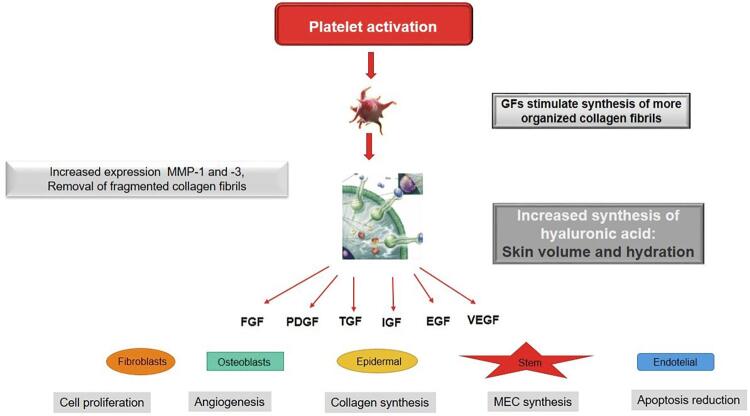
Mechanisms of action of autologous platelet concentrates in facial rejuvenation. After platelets activation, their α granules fuse with the cell membrane, in a process called degranulation. Their growth factors are then secreted, bind to transmembrane receptors on target cells (mesenchymal stem cells, osteoblasts, fibroblasts, endothelial and epidermal cells), activating an intracellular signaling protein that causes the expression of a protein, which, in turn, triggers effects such as cell proliferation, angiogenesis, synthesis of collagen and extracellular matrix components, and reduced apoptosis. With skin aging, fragmented collagen fibrils accumulate, which impairs the growth of new collagen fibers and disrupts the extracellular matrix. Activated platelet aggregates increase the expression of matrix metalloproteases (MMP-1 and -3), stimulating the removal of fragments of collagen fibrils. In addition, they contain several growth factors that stimulate fibroblasts to synthesize new, more organized collagen fibers, besides increasing the synthesis of hyaluronic acid, which binds to water, increasing the skin volume and hydration

To understand the mechanism of action of platelet concentrates in facial rejuvenation, it is necessary to know the platelets. These cells are cytoplasmic fragments of megakaryocytes, formed in the bone marrow, approximately 2 µm in diameter. Platelets contain, in their α granules, protein growth factors with a capital role in hemostasis and wound healing: CTGF (conjunctive tissue growing factor), EGF (epidermal growing factor), FGF-2 and -9 (fibroblast growing factor), IGF-1 (insulin growing factor), PDGF αα (platelet-derived growing factor), PDGF αβ, PDGF ββ, TGF α (transforming growing factor), TGF β1, TGF β2 and VEGF (vascular endothelial growing factor). After platelet exogenous or endogenous activation, these α granules fuse with the cell membrane, in a process called degranulation ( Figure 2 ). These growing factors are then secreted, bind to transmembrane receptors on target cells (undifferentiated mesenchymal cells, osteoblasts, fibroblasts, endothelial cells and epidermal cells), activating an intracellular signaling protein that causes the expression of a protein, which, in turn, triggers effects such as cell proliferation, angiogenesis, synthesis of collagen and extracellular matrix components, and reduced apoptosis.^6 , 16 - 19^ Active secretion of these growth factors by platelets begins 10 minutes after activation, with more than 95% of pre-synthesized growth factors being secreted within 1 hour.^20^

With skin aging, fragmented collagen fibrils accumulate, which impairs the growth of new collagen fibers and disrupts the extracellular matrix.^21^ Activated platelet aggregates increase the expression of matrix metalloproteases (MMP-1 and -3), stimulating the removal of fragments of collagen fibrils. In addition, they contain several growth factors that stimulate fibroblasts to synthesize new, more organized collagen fibers,^22 , 23^ besides increasing the synthesis of hyaluronic acid, which binds to water, increasing the skin volume and hydration^24^ ( Figure 2 ).

### First generation autologous platelet concentrates: PRP

PRP is an autologous plasma preparation with high concentrations of platelets derived from whole blood,^16^ containing more than 800 bioactive molecules.^25 , 26^ The normal concentration of platelets in the blood ranges from 150,000 to 450,000/µL. PRP, by definition, should contain more than 1,000,000 platelets/µL to promote increased tissue healing.^20^ PRP preparations generally have a 4- to 8-fold higher platelet concentration than peripheral blood.^27^ A linear relationship between the concentrations of growth factors and platelets in PRP has been reported.^28^ Although there is still no consensus on the most effective PRP preparation, platelet concentrations higher than 6-fold those of peripheral blood may inhibit healing.^29^ At last instance, the regenerative effect of PRP depends not only on its platelet concentration, but also on the number/type of leukocytes entrapped in the fibrin matrix, and the release of bioactive molecules at the site of injury.^30^

**Figure 3:**
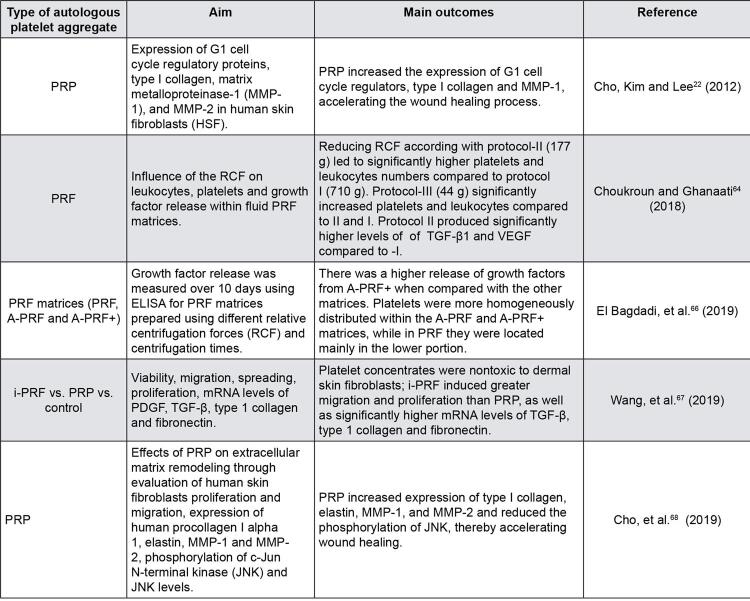
Laboratorial studies evaluating autologous platelet aggregates *PRF = Platelet-rich fibrin; PRP = Platelet-rich plasma; RCF = Relative centrifugation force

PRP contains leukocytes, with catabolic and pro-inflammatory activity, in combination with plasma and growth factors, with anabolic function. These constituents must be in balance so that there is adequate tissue healing and growth, for which the PRP preparation process is fundamental. The two main methods of preparation are the “PRP method” and the “ *buffy coat* ” method.^6^ The latter typically produces PRP with higher platelet concentrations.^31^ There are several commercial kits for preparation of PRP. The composition of the PRP obtained from the different commercial kits varies remarkably. The purpose of PRP preparation methods is to concentrate platelets and to reduce red blood cells. However, the leukocyte levels cannot be neglected. Typically, the kits that employ the “ *buffy coat* “method produce a concentrate containing higher amounts of platelets and red blood cells, but the content of leukocytes is also increased.^31^ Variation in the content of cells and growth factors also depends on the RCF and time of centrifugation employed. Longer and more forceful centrifugation cycles may push platelets down, discharge growth factors and disrupt cellular integrity.^32^ Typically, the bottom layer of red blood cells (RBCs) is discarded, but variable proportions of plasma and buffy coat lead to distinct platelet preparations. These preparations were classified according to the inclusion of the buffy coat (presence of leucocytes) and the use of anticoagulants (formation of fibrin matrix) into 4 categories: 1 – pure platelet-rich plasma (P-PRP); 2 – leucocyte-rich platelet-rich plasma (L-PRP); 3 – pure platelet-rich fibrin (P-PRF); and 4- leucocyte-rich platelet-rich fibrin (L-PRF) .^33^ The last two categories are activated fibrin-based matrices, not a liquid platelet suspension. They are called “second generation” PRP and will be discussed later. Figure 3 shows the main findings of laboratorial studies evaluating different preparations of autologous platelet aggregates.

It is important to avoid contamination with erythrocytes when collecting PRP, as they contain reactive oxygen species, which produce unwanted inflammatory reactions at the site, probably resulting in pain and edema for the patient.^34^ There has been some discussion in the literature about whether the efficacy of the PRP is affected by the inclusion of leucocytes. Despite they might act as antimicrobial agents,^35^ they may also release catabolic cytokines, leading to inflammation and fibrosis, which is more pronounced in the case of neutrophils.^36^ When PRP is employed in soft tissues, there is no need of exogenous activation (with CaCl_2_ or thrombin), since collagen is a natural activator of PRP. When PRP is activated, fibrinogen is transformed in fibrin, creating a fibrin membrane or cloth.^37^

Interestingly, the pH of the platelet concentrate influences its regenerative potential. Preincubation of lysed platelet concentrate at close to pH 5.0 increases its content of available PDGF and its capacity to stimulate fibroblast proliferation. On the other hand, incubation at pH 7.1 increases TGF-β production, which increases collagen production.^38^ However, the applicability of this concept to facial rejuvenation has not been evaluated so far.

The clinical efficacy of PRP depends on the release of bioactive molecules. Therefore, the composition of the PRP is crucial for the clinical effectiveness of the procedures. The main limitations of the PRP research are the imprecise reporting of PRP composition, activation, and dosing, as well as the use of subjective outcome measures. In a systematic review, Frautschi, et al.^39^ (2017) noticed lack of important information in clinical studies evaluating the efficacy of PRP in aesthetic surgery. Most of the studies disregarded either the baseline platelet concentration in the patient’s whole blood or the final platelet concentration in the PRP. This aspect is crucial, since the normal platelet concentrations varies between 150,000-450,000/µL. This 3-fold difference already has an impact in the platelet concentration in the resulting PRP, regardless the technique used for preparation. Like other pharmaceutical drugs, a dose-response relationship has been reported between the platelet concentration and proliferation of fibroblasts, mesenchymal stem cells, and synthesis of type I collagen.^40^ Thus, information regarding the baseline platelet concentration in the whole blood and final platelet concentration in the PRP preparation is crucial.^39^ The use of anticoagulants is not reported in nearly half of the studies.^39^ The type of anticoagulant can have an impact on platelet yield and function.^32^ Another important information involves exogenous activation of PRP. Although there is no consensus on the detriments/benefits of this step, most of the studies (71%) still were found to activate PRP in the time of application. The role of PRP leucocyte concentration is controversial and this information is inconsistently reported. Only 29% of the studies provided this variable.^39^ With these inconsistencies in mind, and considering the PAW classification system,^41^ Frautschi, et al.^39^ (2017) proposed the FIT PAAW classification system. This system is composed of 7 items, each of them containing important information that must be described in clinical studies that evaluate the efficacy of autologous platelet aggregates: (1) **F** orce of centrifugation; (2) **I** teration or sequence of centrifugation; (3) **T** ime of centrifugation; (4) **P** latelet concentration (baseline of patient whole blood and final PRP preparation); (5) **A** nticoagulant use; (6) **A** ctivator use; (7) **W** hite blood cells.

**Figure 4:**
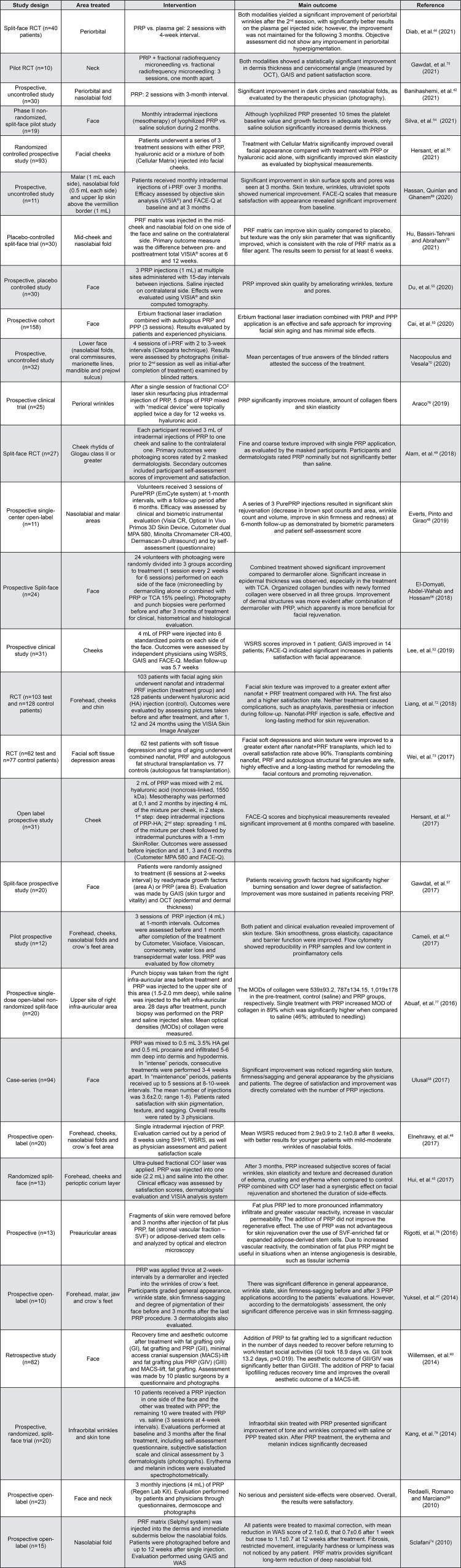
Clinical trials on the use of autologous platelet aggregates for facial rejuvenation

Figure 4 summarizes the evidence for the use of PRP for facial rejuvenation. Twenty-three studies were found. Regarding the periorbital area, (including crow’s feet, dark cycles and infra-orbital wrinkles), seven studies were found.^17 , 42 - 47^ PRP was used as a standalone treatment in most of the cases, or used after CO_2_ laser.^45^ In most of the cases, PRP was applied in two or three sessions, with two/four-week intervals. The studies in general reported favorable results. However, most of the studies employed subjective outcome measures.

In all studies that evaluated PRP for treatment of nasolabial folds, significant improvement was reported ^42 , 43 , 46 , 48^ , both by self-assessment or evaluation by physicians, as well as by biometric evaluation. In one of the studies, PRP was injected only once;^46^ however, treatment used to be performed in two or three sessions, with a one-month interval. Patients were followed up to six months.^48^

Regarding the use of PRP for the treatment of the cheeks and malar area, eight studies were found.^43 , 45 - 47 , 49 - 52^ In general, the beneficial results reported were less evident than those noticed for the nasolabial folds. Hersant, et al.^50^ (2021) did not find any beneficial effect of PRP when used alone, but only when employed together with hyaluronic acid (HA). The association between PRP and HA, applied in two steps (mesotherapy and dermaroller) was also proven to be efficient in the study by Hersant et al.^51^ (2017). Alam, et al.^49^ (2018) reported that patients and dermatologists rated PRP nominally but not significantly better than saline. Lee, et al.^52^ (2019) reported that WSRS scores improved in one patient, GAIS improved in 14 patients and FACE-Q indicated significant increases in patients’ satisfaction with facial appearance after 4 mL of PRP were injected in six standardized points of the face. Hui, et al.^45^ (2017) found a synergistic effect between PRP and CO_2_ laser for facial rejuvenation.

Eight studies evaluated the whole face^53 - 60^ after treatment with PRP. In some of them, PRP was combined with other treatments/actives, such as erbium fractioned laser;^53^ HA gel^58^ , dermaroller microneedling^56^ or lipofiller.^60^ In all cases, the association was reported to provide beneficial results. PRP provided significantly better improvement and less side effects when compared with readymade growth factors.^57^ A split-face study showed that three PRP injections at multiple sites administered with 15-day intervals improved skin quality by ameliorating wrinkles, texture and pores.^55^ In a recent split-face study, monthly intradermal injections (mesotherapy) of lyophilized PRP were compared to saline solution for two months. Although lyophilized PRP presented 10 times the platelet baseline value and growth factors in adequate levels, only saline solution significantly increased dermis thickness.

A recent systematic review evaluated the safety and effectiveness of PRP for skin aging. In total, 24 studies, with 480 patients, were included. As monotherapy, PRP induced modest improvement in facial skin texture, appearance, and lines, besides improving pigmentation and fine lines, based on physician assessment. The degree of satisfaction of patients was high, although the degree of improvement was in general lower than 50% and the duration of the effect was uncertain. The degree of evidence is limited by heterogeneity in PRP preparation and administration, and lack of standardization in outcome measures. Moreover, only half of the studies employed “blind” examiners, which might have led to overestimation of effectiveness. The authors concluded that more high-quality trials with appropriate follow-up are necessary to provide appropriate evidence that may help to improve the treatment regimens. Several aspects should be considered when future clinical trials evaluating PRP are to be designed, such as the patients’ characteristics (age, gender, history of sun exposure, ethnicity) that best predict a favorable response; the optimal number of sessions and the interval between them; the characteristics of the studies (quantification of the main parameters of PRP growth factors, longitudinal evaluation, examiners blinding); development of better instruments to evaluate skin aging.^5^

A recent review, including six articles, reported that PRP has been used effectively not only as an adjuvant therapy, but also as a standalone treatment for melasma. Among the growth factors present in PRP, TGF-β plays a central role in the treatment of melasma, since it reduces signal transduction of microphthalmia-induced transcription factor, thereby decreasing tyrosinase and tyrosinase-related proteins. Moreover, PRP also induces collagen synthesis, improving the quality and texture of the skin.^14^

One of the most frequent complaints of the patients undergoing treatment with PRP is pain during application, especially when the treatment is performed by multiple injections. It has been reported that covering the area of application of PRP with a cooled (20ºC) hydrogel dressing for 20 minutes before and after PRP injection reduces pain and edema by needle picking and accelerates patient recovery and overall appearance of the skin straight after the procedure.^61^ Moreover, a thermosensitive formulation able to embed PRP and growth factors that stays liquid when the temperature is lower than 20ºC, but becomes a gel when the temperature exceeds 35ºC (when the product touches the skin), was developed. This thermosensitive gel formulation was named “medical device” and allows storage of platelets and growth factors for seven days, maintaining their full activity.

Although PRP has been reported to be used for the treatment of infraorbital hyperpigmentation and also for treatment of post inflammatory hyperpigmentation, especially seen after peeling or laser applications ^62^ , this is controversial, since there are reports showing increased pigmentation when it is applied over the pigmented skin lesions that were present before the application.^63^ This is the reason why it has been reported that PRP should not be used to treat post inflammatory hyperpigmentation.^63^

PRP preparation is difficult due to the requirement of double centrifugation.^5^ In addition, the need to use anticoagulant might impair the healing process, due to the inhibition of the coagulation process.^7^ To overcome these limitations, platelet-rich fibrin (PRF), an autologous platelet aggregate of “second generation,” was developed by Choukroun, et al.^8^ (2001).

#### Second and third generation autologous platelet concentrates: PRF and i-PRF

PRF is obtained after a single centrifugation, without the need of using anticoagulants.^8^ The resulting product contains different cell types (platelets, leucocytes, erythrocytes), an extracellular fibrin matrix and several bioactive molecules (primarily growth factors). Depending on the type of tube used for collecting the blood and on the protocol for centrifugation, PRFs in the form of liquid or solid gel can be obtained. The solid forms obtained using glass tubes have been used in plastic and bucomaxillofacial surgeries.^7 , 9^

In 2014, a fluid (injectable) form of PRF (called i-PRF; “third generation”) was developed, by modification of the RCF.^10^ Reducing the RCF and the time of centrifugation and using plastic tubes (to reduce the coagulation time), the time required for the coagulation of the fibrin could be slower in the initial periods, generating a product containing fibrinogen and thrombin, which remains fluid for around 20 minutes after centrifugation, before the fibrin matrix is formed. This makes the product proper to be employed for facial rejuvenation.

By employing lower RCFs (enough to separate erythrocytes from platelets), the characteristics of the PRF are improved. The numbers of platelets and leucocytes and the concentrations of growth factors in the fibrin matrix are increased. Moreover, platelets and cytokines are entrapped in the fibrin matrix after the injection, leading to a slow and gradual release of growth factors along time.^64 , 65^ In the study by Choukroun and Ghanaati ^64^ , plasma was centrifuged using RCFs of 710 *g* , 177 *g* and 44 *g* for 8 min. A higher concentration of platelets and leucocytes was found in the iPRF when the RCF of 44 *g* was employed, while higher concentrations of growth factors (VEGF and TGF-β1) were found with 177 *g* . In another study, it was evaluated the pattern of platelets distribution and the release of growth factors (EGF, VEGF and TGF-β1) along time from three PRF matrixes, produced from distinct RCFs and times of centrifugation: PFR (708 *g* , 12 min), A-PRF (advanced; 208 *g* ; 14 min), A-PRF+ (advanced+; 208 *g* ; 8 min). A-PRF+ led to a higher release of growth factors when compared with the other matrices. In addition, platelets had a more homogeneous distribution in the A-PRF and A-PRF+ matrices, while in the PRF matrix, they were located mainly in the lower portion^66^ ( Figure 3 ).

Experiments with human dermal skin fibroblasts showed greater cell migration and proliferation, as well as higher levels of m-RNA for type I collagen, TGF-β and fibronectin, besides a higher capacity to induce the synthesis of collagen matrix in the presence of i-PRF when compared with PRP^67^ ( Figure 3 ). PRP reduces the phosphorylation of JNK, thereby accelerating would healing.^68^

Figure 4 shows clinical trials on the use of autologous platelet aggregates for facial rejuvenation. Only six clinical trials evaluated the use of PRF and i-PRF for facial rejuvenation ^69 - 74^ , while most of the studies evaluated PRP.^42 - 60 , 75 - 79^ Regarding the studies evaluating PRF or iPRF, two of them found beneficial results when PRF was combined with nanofat.^72 - 73^ When used alone, PRF matrix provided significant long-term reduction of deep nasolabial fold.^70 , 74^ It has been reported, using i-PRF with low RCF (combination of 60 *g* for 3 min and 208 *g* for 5 min), good results for the rejuvenation of the lower third of the face (nasolabial fold and labial commissure) after an intradermal application.^71^ In another study, the effect of three monthly intradermal injections of i-PRF (low RCF 60 *g* , 3 min) in three facial regions was evaluated: malar area, nasolabial fold, and region above the vermilion of the upper lip. An improvement in skin texture, pores, wrinkles, as well as patient satisfaction was observed after three months.^69^ However, additional studies are needed to establish the centrifugation protocol that leads to the best clinical effects. In addition, more high-quality trials with appropriate follow-up are necessary to provide appropriate evidence that may help to improve the treatment regimens.

## Conclusion

Autologous platelet aggregates for skin rejuvenation are safe and well tolerated. PRP, the first-generation product, is more studied in the literature, with several clinical trials and case series, whose results have been complied in a systematic review.^5^ The results, in general, are favorable, but the quality of the studies is low and additional studies are required. The second and third generation products, PRF and i-PRF, respectively, are easier to be obtained and, at least *in vitr* o, seem to induce greater collagen production than PRP,^67^ especially under lower RCFs.^64^ However, only a few clinical trials evaluating these products are available to date.

More high-quality trials with appropriate follow-up are necessary to provide appropriate evidence that may help to improve the treatment regimens with autologous platelet aggregates. Several aspects should be considered when future clinical trials evaluating PRP are to be designed, such as the patients’ characteristics that best predict a favorable response, the optimal number of sessions and the interval between them, the characteristics of the studies and the development of better instruments to evaluate skin aging.
